# Inhibition of microglia overactivation restores neuronal survival in a mouse model of CDKL5 deficiency disorder

**DOI:** 10.1186/s12974-021-02204-0

**Published:** 2021-07-08

**Authors:** Giuseppe Galvani, Nicola Mottolese, Laura Gennaccaro, Manuela Loi, Giorgio Medici, Marianna Tassinari, Claudia Fuchs, Elisabetta Ciani, Stefania Trazzi

**Affiliations:** Department of Biomedical and Neuromotor Sciences, University of Bologna, Piazza di Porta San Donato 2, 40126 Bologna, Italy

**Keywords:** CDKL5, Neuroinflammation, Neuronal survival, Luteolin

## Abstract

**Background:**

CDKL5 deficiency disorder (CDD), a severe neurodevelopmental disorder characterized by early onset epilepsy, intellectual disability, and autistic features, is caused by mutations in the *CDKL5* gene. Evidence in animal models of CDD showed that absence of CDKL5 negatively affects neuronal survival, as well as neuronal maturation and dendritic outgrowth; however, knowledge of the substrates underlying these alterations is still limited. Neuroinflammatory processes are known to contribute to neuronal dysfunction and death. Recent evidence shows a subclinical chronic inflammatory status in plasma from CDD patients. However, to date, it is unknown whether a similar inflammatory status is present in the brain of CDD patients and, if so, whether this plays a causative or exacerbating role in the pathophysiology of CDD.

**Methods:**

We evaluated microglia activation using AIF-1 immunofluorescence, proinflammatory cytokine expression, and signaling in the brain of a mouse model of CDD, the *Cdkl5* KO mouse, which is characterized by an impaired survival of hippocampal neurons that worsens with age. Hippocampal neuron survival was determined by DCX, NeuN, and cleaved caspase-3 immunostaining in *Cdkl5* KO mice treated with luteolin (10 mg/kg), a natural anti-inflammatory flavonoid. Since hippocampal neurons of *Cdkl5* KO mice exhibit increased susceptibility to excitotoxic stress, we evaluated neuronal survival in *Cdkl5* KO mice injected with NMDA (60 mg/kg) after a 7-day treatment with luteolin.

**Results:**

We found increased microglial activation in the brain of the *Cdkl5* KO mouse. We found alterations in microglial cell morphology and number, increased levels of AIF-1 and proinflammatory cytokines, and activation of STAT3 signaling. Remarkably, treatment with luteolin recovers microglia alterations as well as neuronal survival and maturation in *Cdkl5* KO mice, and prevents the increase in NMDA-induced cell death in the hippocampus.

**Conclusions:**

Our results suggest that neuroinflammatory processes contribute to the pathogenesis of CDD and imply the potential usefulness of luteolin as a treatment option in CDD patients.

**Supplementary Information:**

The online version contains supplementary material available at 10.1186/s12974-021-02204-0.

## Background

The cyclin-dependent kinase-like 5 gene (*CDKL5*), located on the short arm of the X chromosome [1], encodes for a serine/threonine kinase that is highly expressed in the brain [2]. Genetic mutations of this gene cause absence of a functional CDKL5 protein, resulting in a severe neurodevelopmental encephalopathy named CDKL5 deficiency disorder (CDD; OMIM 300203, 300672). This disorder is associated with early onset epilepsy and severe cognitive, motor, visual, and sleep disturbances [3–5]. An animal model of CDD, the *Cdkl5* knockout (KO) mouse [6–9], recapitulates, to varying degrees, the underlying molecular and behavioral defects of the human disease. Interestingly, mouse models of CDD do not have recurrent epileptic seizures, but nonetheless demonstrate significant behavioral deficits: learning and memory, motor control and social interaction deficits, visual impairments, and sleep disturbances [6–14].

Changes in neuronal morphology such as dendritic branching and stability of dendritic spines have been consistently reported in *Cdkl5* KO mice [6, 7]. *Cdkl5* KO mice showed a reduction in dendritic length of cortical and hippocampal pyramidal neurons [6, 11, 15, 16] and changes in the maturation and stability of dendritic spines, as well as in the density of PSD-95 dendritic clusters in several brain structures [10, 15–18].

Interestingly, recent evidence has shown that CDKL5, in addition to neuronal maturation and dendritic outgrowth, also affects neuronal survival [11, 19, 20]. *Cdkl5* KO mice are characterized by an increased rate of apoptotic cell death in the hippocampal dentate gyrus that causes a reduction in the final number of granule neurons [11] and by accelerated neuronal senescence/death during aging [21]. Moreover, hippocampal neurons of *Cdkl5* KO mice exhibit increased susceptibility to neurotoxic/excitotoxic stress [19, 20], indicating that absence of Cdkl5 increase neuronal vulnerability.

Neuroinflammatory processes are known to contribute to neuronal dysfunction and death. When overactivated in response to neuronal damage and genetic or environmental factors, microglia, the brain macrophages [22], cause widespread damage to neighboring neurons. Indeed, reactive microglia kill neurons by producing neurotoxic factors and proinflammatory molecules such as tumor necrosis factor-α (TNF-α) and interleukin-1β and interleukin-6 (IL-1β, IL-6) [23, 24]. Remarkably, overactivated microglia have been described in several neurodegenerative diseases such as Alzheimer’s, Parkinson’s, and Huntington’s diseases and amyotrophic lateral sclerosis [25–27], suggesting that active neuroinflammation may account for the compromised neuronal survival observed in these pathologies. Interestingly, active neuroinflammation could account for the compromised brain development observed in neurodevelopmental disorders such as Down syndrome, autism spectrum disorders (ASD), and Rett syndrome [28–30], by damaging synaptic connectivity.

Recently, a major cytokine dysregulation proportional to clinical severity, inflammatory status, and redox imbalance was evidenced in plasma from CDD patients [31, 32], suggesting a subclinical chronic inflammatory status in children affected by this pathology. However, to date, it is unknown whether such an inflammatory state is even mirrored at the cerebral level and whether it can contribute to the pathophysiology of CDD.

Here, we show evidence of a microglia overactivation status in the brain of *Cdkl5* KO mice, characterized by alterations in microglia cell number/morphology and increased proinflammatory gene expression. We found that microglia overactivation is already present in the postnatal period in *Cdkl5* KO mice and worsens during aging. Importantly, by restoring microglia alterations, treatment with luteolin, a natural anti-inflammatory flavonoid, counteracts hippocampal neuron cell death in both adult and aged *Cdkl5* KO mice, and rescues NMDA-induced excitotoxic damage in *Cdkl5* KO mice. These findings highlight new insights into the CDD pathophysiology and provide the first evidence that therapeutic approaches aimed at counteracting neuroinflammation could be beneficial in CDD.

## Materials and methods

### Colony and husbandry

The mice used in this work derived from the *Cdkl5* null strain in the C57BL/6N background developed in [6] and backcrossed in C57BL/6J for three generations. Mice for experiments were produced by crossing *Cdkl5* +/− females with *Cdkl5* -/Y males and *Cdkl5* +/− females with +/Y males [6]; animals were karyotyped by PCR on genomic DNA as previously described [6], and littermate controls were used for all experiments. P0, postnatal day zero, was designated as the day of birth, and 24 h later, mice were considered 1-day-old animals (P1). Mice were housed three to five per cage and maintained in a temperature- (23° C) and humidity-controlled environment with a standard 12 h light/dark cycle, and provided with standard mouse chow and water *ad libitum.* Animals’ day-to-day health and comfort were monitored by the veterinary service. All possible efforts were made to minimize suffering and the number of animals used. Experiments were carried out on a total of 139 *Cdkl5* KO mice (*Cdkl5* +/Y, n=4; *Cdkl5* -/Y, n=4; *Cdkl5* +/+, n= 43; *Cdkl5* +/-, n= 88).

### Treatments

All treatments were performed in the animal house at the same hour of the day.

#### Luteolin treatment

The dose of luteolin was chosen based on [33]. Mice were intraperitoneally (i.p.) injected daily with vehicle (2% DMSO in saline) or luteolin (10 mg/kg in saline; Tocris) for 7 or 20 days. The day following the last treatment, mice were sacrificed for histological and Western blot analyses.

#### Stattic treatment

The dose of Stattic was chosen based on [34]. Mice were i.p. injected with vehicle (2% DMSO + 30% polyethylene glycol) or Stattic (20 mg/Kg; Sigma-Aldrich) every other day for 7 days for a total of four injections. Animals were sacrificed the day following the last treatment for histological analyses.

#### Paracetamol/acetaminophen treatment

The dose of acetaminophen (APAP) was chosen based on [28]. Mice were daily i.p. injected with vehicle (2% DMSO in saline) or APAP (100 mg/kg; Sigma-Aldrich) for 7 days, and sacrificed the day following the last treatment.

#### NMDA treatment

Mice were treated with an intraperitoneal injection of NMDA (60 mg/kg; Sigma-Aldrich) in phosphate-buffered saline (PBS) after 7 days of luteolin treatment. Animals were sacrificed 24 h or 8 days after NMDA injection, and immunohistochemical analysis was assessed as described below. Seizure grades were scored according to [35] and recorded during a 1-h observation period. NMDA-induced seizures were scored as follows: 0—no abnormalities; 1—exploring, sniffing, and grooming ceased, with mice becoming motionless; 2—forelimb and/or tail extension, appearance of rigid posture; 3—myoclonic jerks of the head and neck with brief twitching movements, or repetitive movements with head-bobbing or “wet-dog shakes”; 4—forelimb clonus and partial rearing, or rearing and falling; 5—forelimb clonus, continuous rearing, and falling; 6—tonic-clonic movements with loss of posture tone, often resulting in death.

#### TATκ-GFP-CDKL5 protein treatment

Brain sections processed for AIF-1 immunohistochemistry were derived from animals used in [36]. Briefly, 6-month-old wild-type (+/Y) and *Cdkl5* (-/Y) mice were implanted subcutaneously with a programmable pump (IPRECIO, Primetech, Japan) equipped with a refillable reservoir. The pump was connected to a catheter implanted in the carotid artery. The IPRECIO reservoir (130 μl) was filled with either TATκ-GFP-CDKL5 or TATκ-GFP. A 10-day infusion protocol was programmed as follows: 2 daily (1 in the morning and 1 in the evening) boluses (20 μl each corresponding to 50 ng of protein) were administered at 10 μl/h with a low constant release (0.4 μl/h) during the rest of the day to prevent catheter occlusion. Every 2 or 3 days, mice were briefly anesthetized to refill the IPRECIO reservoir (transdermal injection) with fresh solutions.

### Histological and immunohistochemical procedures

Animals were anesthetized with 2% isoflurane (in pure oxygen) and sacrificed through cervical dislocation. Brains were quickly removed and cut along the midline. While right hemispheres were quickly frozen and used for Western blot analyses, left hemispheres were fixed via immersion in 4% paraformaldehyde (100 mM phosphate buffer, pH 7.4) for 48 h, kept in 15–20% sucrose for an additional 24 h, and then frozen with cold ice. Hemispheres were cut with a freezing microtome into 30-μm-thick coronal sections and processed for immunohistochemistry procedures as described below. Brain sections from *Emx1* KO mice used in [37] were processed for AIF-1 and NeuN immunohistochemistry.

#### AIF-1, NeuN, GFAP, GAD67, and Ki-67 immunohistochemistry

One out of every eight free-floating sections from the hippocampal formation was incubated with one of the following primary antibodies: rabbit polyclonal anti-AIF-1 antibody (1:300; Thermo Fisher), mouse monoclonal anti-NeuN antibody (1:250; Merck Millipore), rabbit polyclonal anti-GFAP antibody (1:400; Abcam), mouse monoclonal anti-GAD67 antibody (1:500; Merck Millipore), or rabbit monoclonal Ki-67 antibody (1:100; Thermo Scientific). Sections were then incubated for 2 h at room temperature with a Cy3-conjugated anti-rabbit secondary antibody (1:200, Jackson ImmunoResearch Laboratories, Inc.) for AIF-1, GFAP, and Ki-67 immunohistochemistry, and with a Cy3-conjugated anti-mouse secondary antibody (1:200, Jackson ImmunoResearch Laboratories, Inc.) for GAD67 and NeuN immunohistochemistry. Nuclei were counterstained with Hoechst-33342 (Sigma-Aldrich).

#### Cleaved caspase-3 staining

For cleaved caspase-3 immunofluorescence, one out of every six sections from the hippocampal formation was incubated overnight with a rabbit polyclonal anti-cleaved caspase-3 antibody (1:200; Cell Signaling Technology). The following day, the sections were washed and incubated with an HRP-conjugated anti-rabbit secondary antibody (1:200; Jackson ImmunoResearch, Inc.). Detection was performed using the TSA Cyanine 3 Plus Evaluation Kit (Perkin Elmer), and nuclei were counterstained with Hoechst 33342 (Sigma-Aldrich).

#### DCX immunohistochemistry

One out of every six free-floating sections from the hippocampal formation was incubated overnight with a goat polyclonal anti-doublecortin antibody (DCX, 1:100, Santa Cruz Biotechnology, Inc.). The following day, the sections were washed and incubated with a biotinylated anti-goat IgG secondary antibody (1:200, Vector BioLabs, Malver, PA, USA) for 2 h and thereafter for 1 h with the VECTASTAIN®ABC kit (Vector BioLabs). Detection was performed using DAB kit (Vector BioLabs).

### Image acquisition and measurements

Fluorescence images were taken with an Eclipse TE 2000-S microscope equipped with a DS-Qi2 digital SLR camera (Nikon Instruments Inc.). A light microscope (Leica Mycrosystems) equipped with a motorized stage and focus control system and a color digital camera (Coolsnap-Pro, Media Cybernetics) were used for neuronal tracing and to take bright field images. Measurements were carried out using the Image Pro Plus software (Media Cybernetics, Silver Spring, MD, USA).

#### Cell density

The number of AIF-1-positive cells in the hippocampus and somatosensory cortex was manually counted using the point tool of the Image Pro Plus software (Media Cybernetics, Silver Spring, MD, USA), and cell density was established as AIF-1-positive cells/mm^3^. The density of Hoechst-positive nuclei, neurons (NeuN-positive), apoptotic cells (cleaved caspase-3-positive), inhibitory interneurons (GAD67-positive cells), and astrocytes (GFAP-positive cells) in the CA1 layer were manually counted and expressed as cells/mm^3^. Ki-67-positive cells were counted in the subgranular zone of the dentate gyrus and expressed as number of cells/mm.

#### Morphometric microglial cell analysis

Starting from 20× magnification images of AIF-1-stained hippocampal and cortical slices, AIF-1 positive microglial cell body size was manually drawn using the Image Pro Plus measurement function and expressed in μm^2^. The roundness index of each microglia cell was calculated as reported in [38] with the equation: roundness = 4A/πM^2^, where A is the area and M is the length of the major axis of each microglia cell’s soma. Approximately 120 microglia cells were analyzed from each sample.

#### Number and neuronal tracing of doublecortin (DCX)-positive cells

DCX-positive cells were counted in the subgranular zone and in the granular layer of the dentate gyrus and expressed as number of neurons/100 μm. Dendritic trees of newborn DCX-positive granule neurons (15–20 per animal) were traced using custom-designed software for dendritic reconstruction (Immagini Computer, Milan, Italy), interfaced with Image Pro Plus. The dendritic tree was traced live, at a final magnification of 500×. The program provides the total dendritic length once the reconstruction of the entire dendritic tree is finished.

### Microglia cell isolation

Microglial cells were isolated following the protocol published in [39]. Briefly, wild-type (+/+) and *Cdkl5* KO (+/-) female mice were anesthetized with 2% isoflurane (in pure oxygen) and transcardial perfused with PBS. Brains were transferred to ice-cold phosphate-buffered saline (PBS; without Ca2+ and MG2+, with NaHCO_3_, 0.75 g/l, Hepes buffer 10 mM, pH 7.4), freed of meninges, minced in serum-free Dulbecco Modified Eagle Medium (DMEM) containing 0.25% trypsin (T9201; Sigma-Aldrich, St. Louis, MO, USA) and 0.02% EDTA, and incubated at 37°C for 60 min. Enzymatic digestion was blocked by adding DMEM supplemented with 10% heat-inactivated FBS. After centrifugation (2000 rpm at 4°C for 5 min), samples were incubated with a DNAse I solution (serum-free DMEM containing 40 μg/ml DNAse Type I, D5025; Sigma-Aldrich, St. Louis, MO, USA) for 15 min at 37°C. Samples were then centrifuged, transferred to ice-cold DMEM, and sieved through a nylon mesh 40-μm pore size (Corning Cell Strainer). DMEM (21.4 ml) with sieved tissue derived from 2 mice brain was mixed with 8.6 ml of cold isotonic Percoll (Formerly GE Healthcare Life Sciences; Fisher scientific) in PBS and centrifuged (2000 rpm at 4°C for 20 min). Pellet was washed in PBS and finally resuspended in 1 ml of TRI reagent (Sigma-Aldrich, St. Louis, MO, USA) and stored at −80°C or processed for RNA extraction. For Western blotting analyses, protein extracts were prepared from microglial cells purified from 4 to 6 mouse brains.

### RNA isolation and RT-qPCR

Microglia cell RNA isolation was performed using the Direct-zol RNA MiniPrep kit (Zymo Research), and cDNA synthesis was achieved with 1 μg of total RNA using iScript™ Advanced cDNA Synthesis Kit (Bio-Rad, Hercules, CA, USA) according to the manufacturer’s instructions. Real-time PCR was performed using SsoAdvanced Universal SYBR Green Supermix (Bio-Rad) in an iQ5 Real-Time PCR Detection System (Bio-Rad). We used primer pairs (table S1) that gave an efficiency close to 100%. GAPDH (glyceraldehyde 3-phosphate dehydrogenase) was used as a reference gene for normalization in the qPCR. Each biological replicate was run in triplicates. Relative quantification was performed using the ΔΔCt method.

### Western blotting

For the preparation of total cell extracts, tissue samples were homogenized in RIPA buffer (50 mMTris–HCl, pH 7.4, 150 mM NaCl, 1% Triton-X100, 0.5% sodium deoxycholate, 0.1% SDS) supplemented with 1 mM PMSF and 1% protease and phosphatase inhibitor cocktail (Sigma-Aldrich). Protein concentration was determined using the Bradford method [40]. Equivalent amounts (50 μg) of protein were subjected to electrophoresis on a 4–12% Mini-PROTEAN® TGX™ Gel (Bio-Rad) and transferred to a Hybond ECL nitrocellulose membrane (GE Healthcare Bio-Science). The following primary antibodies were used: rabbit polyclonal anti-GAPDH (1:5000, Sigma-Aldrich), rabbit polyclonal anti-STAT3 (1:1000, Sigma-Aldrich), rabbit polyclonal anti-phospho-STAT3 (1:1000, Cell Signaling Technology), rabbit polyclonal anti-AIF-1 antibody (1:1000; Thermo Fisher), and rabbit polyclonal anti-BDNF (1:500, Santa Cruz Biotechnology). An HRP-conjugated goat anti-rabbit IgG (1:5000, Jackson ImmunoResearch Laboratories) secondary antibody was used. P-STAT3, STAT3, and BDNF Western blot analyses were performed on protein extracts of four animals per experimental group. Repeated measurements of the same samples were performed running from two to four different gels. The signal of one sample (internal control) was used to perform a relative analysis of the antigen expression of each sample on the same gel. We considered the control signal as 100 and assigned a value to the other sample as a percentage of the control. Data analysis was performed by averaging the signals obtained in two to four gels for each individual sample. The densitometric analysis of digitized Western blot images was performed using Chemidoc XRS Imaging Systems and the Image LabTM Software (Bio-Rad), this software automatically highlighting any saturated pixels of the Western blot images in red. Images acquired with exposition times that generated protein signals out of a linear range were not considered for the quantification.

### Statistical analysis

Statistical analysis was performed with GraphPad Prism (version 7). Values are expressed as means ± standard error (SEM). The significance of results was obtained using two-tailed unpaired t-test and one-way or two-way ANOVA followed by Fisher’s LSD post hoc test as specified in the figure legends. A probability level of p < 0.05 was considered to be statistically significant. The confidence level was taken as 95%.

## Results

### Increased microglial activation in the brain of *Cdkl5* KO mice

**Fig. 1:**
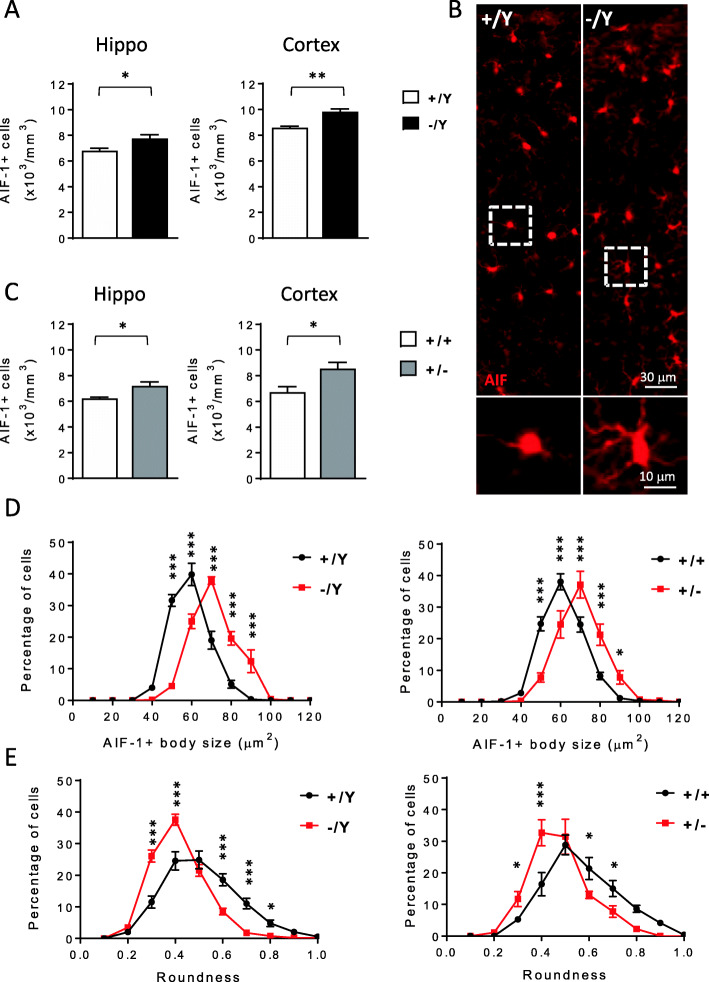
Increased microglial activation in the brain of *Cdkl5* KO mice. **A**, **C** Quantification of AIF-1-positive cells in hippocampal (Hippo) and somatosensory cortex (Cortex) sections from 3-month-old male (+/Y n=4, -/Y n=4; **A**), and female (+/+ n=5, +/- n=6; **C**) *Cdkl5* KO mice. **B** Representative fluorescence images of cortical sections processed for AIF-1 immunohistochemistry of a wild-type (+/Y) and a *Cdkl5* KO mouse (-/Y). The dotted boxes in the upper panels indicate microglial cells shown in magnification in lower panels. **D** Distribution analysis of microglial cell soma area in the somatosensory cortex of 3-month-old male (+/Y n=4, -/Y n=4 on the left), and female (+/+ n=4, +/- n=6 on the right) *Cdkl5* KO mice, showing a shift to larger cell body sizes in the absence of *Cdkl5*. **E** Distribution analysis of microglial cell circularity (roundness) in the somatosensory cortex of *Cdkl5* KO mice as in **D**. In *Cdkl5* KO mice, microglial cells are more irregularly shaped (lower roundness index) showing a left-shifted distribution compared to that of wild-type mice. The results in **A** and **C** are presented as means ± SEM. * p<0.05; ** p<0.01 (two-tailed Student’s t-test). The results in **D** and **E** are presented as means ± SEM. * p<0.05; ***p<0.001 (Fisher’s LSD test after two-way ANOVA)

To investigate whether inflammatory processes could be involved in the pathophysiology of CDD, we counted the number and analyzed the morphology of microglia (AIF-1-positive cells) in the hippocampus and cortex of male (-/Y) and female (+/-) *Cdkl5* KO mice and wild-type (+/Y, +/+) littermates. We found an increase in the number of microglial cells in both the analyzed brain regions of -/Y and +/- *Cdkl5* KO mice in comparison with their +/Y and +/+ counterparts (Fig. 1A–C). Moreover, microglial cells in *Cdkl5* KO mice presented an enlarged body size (Fig. 1D and Fig S1A,B) and reduced roundness of the cell body (Fig. 1E) compared to wild-type counterparts (Fig. 1B, D, E). Together, these data indicate that in the absence of *Cdkl5*, microglia adopted a bigger, more irregular soma shape, typical of a state of activation [41].

**Fig. 2:**
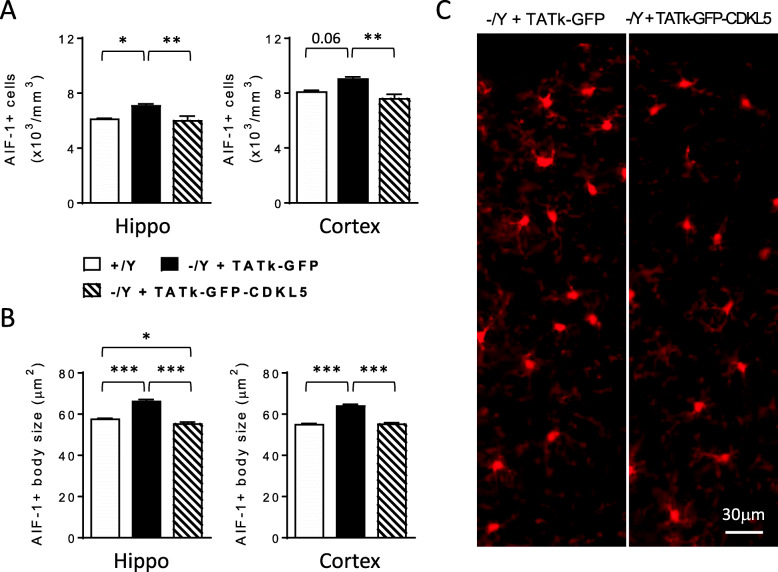
Effect of TATκ-GFP-CDKL5 administration on microglial cell activation in *Cdkl5* KO mice. **A** Quantification of AIF-1-positive cells in hippocampal (Hippo) and somatosensory cortex (Cortex) sections from 6-month-old wild-type (+/Y n=2) mice and *Cdkl5* -/Y mice treated with TATκ-GFP (n= 5) or TATκ-GFP-CDKL5 (n= 5) protein as described in [36]. **B** Mean microglia cell body size in the hippocampus and somatosensory cortex of *Cdkl5* KO mice as in **A**. Data in **A** and **B** are presented as means ± SEM. * p<0.05; ** p<0.01; ***p<0.001 (Fisher’s LSD after one-way ANOVA). **C** Examples of cortical sections processed for AIF-1 immunostaining of a *Cdkl5* -/Y mouse treated with TATκ-GFP or TATκ-GFP-CDKL5 as in **A**

Importantly, replacement of CDKL5 protein through a systemic injection of a TATκ-GFP-CDKL5 fusion protein [36] reversed microglial activation in *Cdkl5* KO mice. We found a lower number of microglial cells (Fig. 2A) with a smaller body size (Fig. 2B) in the hippocampus and cortex of TATκ-GFP-CDKL5-treated *Cdkl5* -/Y mice compared to *Cdkl5* -/Y mice treated with a TATκ-GFP control protein, indicating the reversibility of the inflammatory phenotype due to the absence of Cdkl5.

### Non-cell autonomous microglial activation in the absence of *Cdkl5*

**Fig. 3:**
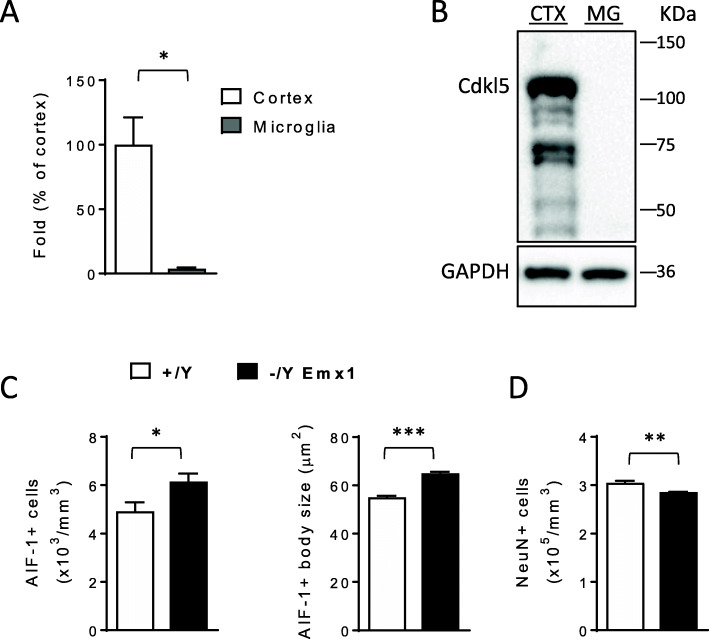
Non-cell autonomous microglial activation in the absence of Cdkl5. **A** Expression of *Cdkl5* mRNA in cortex of 3-month-old *Cdkl5* +/+ mice (n=3) and microglial cells purified from 3-month-old *Cdkl5* +/+ mice (n=3). Data are given as a percentage of *Cdkl5* cortical expression. **B** Example of immunoblot showing Cdkl5 and GAPDH levels in extracts from somatosensory cortex (CTX) of a wild-type *Cdkl5* +/+ mouse and from microglial cells (MG) purified from *Cdkl5* +/+ mice (n=4). **C** Quantification of AIF-1-positive cells (on the left) and mean cell body size (on the right) of microglial cells in hippocampal sections of wild-type (+/Y; n=4) and *Emx1* KO (-/Y Emx1; n=5) mice. **D** Quantification of NeuN positive cells in CA1 layer of hippocampal sections of wild-type (+/Y; n=3) and *Emx1* KO (-/Y Emx1; n=3) mice. The results in **A**, **C**, and **D** are presented as means ± SEM. * p<0.05; **p< 0.01; ***p<0.001 (two-tailed Student’s t-test)

In order to investigate whether microglial activation in *Cdkl5* KO mice is a cell-autonomous effect, we first evaluated Cdkl5 expression levels in purified microglial cells. Isolation of highly enriched microglial cells was confirmed by the high levels of microglia specific markers (AIF-1 and CD11b) and low levels of the neuronal marker (NeuN) in microglia extracts in comparison with cortical extracts from wild-type mice (Fig. S2). Regarding Cdkl5 expression, real time and western blot analyses showed very low *Cdkl5* mRNA levels (Fig. 3A) and undetectable protein levels (Fig. 3B) in microglial cells compared to cortical extracts of wild-type mice, suggesting that Cdkl5 function is of minor relevance in these cells. Next, we evaluated the microglia activation status in *Emx1* KO mice, a conditional *Cdkl5* KO mouse model carrying *Cdkl5* deletion only in the excitatory neurons of the forebrain, but not in microglial cells [6, 37]. Similarly to *Cdkl5* KO mice, activation of microglial cells, increased number and body size of microglial cells (Fig. 3C), was present in the hippocampus of *Emx1* KO mice, suggesting a non-cell autonomous microglial overactivation in the absence of Cdkl5, probably caused by alterations at the neuronal level. We found that *Emx1* KO mice, similarly to *Cdkl5* KO mice [21], showed a decreased number of NeuN-positive cells in the hippocampal CA1 layer (Fig. 3D), suggesting that neuronal loss could underlie microglia overactivation in *Emx1* KO mice.

### Treatment with luteolin inhibits microglia overactivation in *Cdkl5* +/- mice

**Fig. 4:**
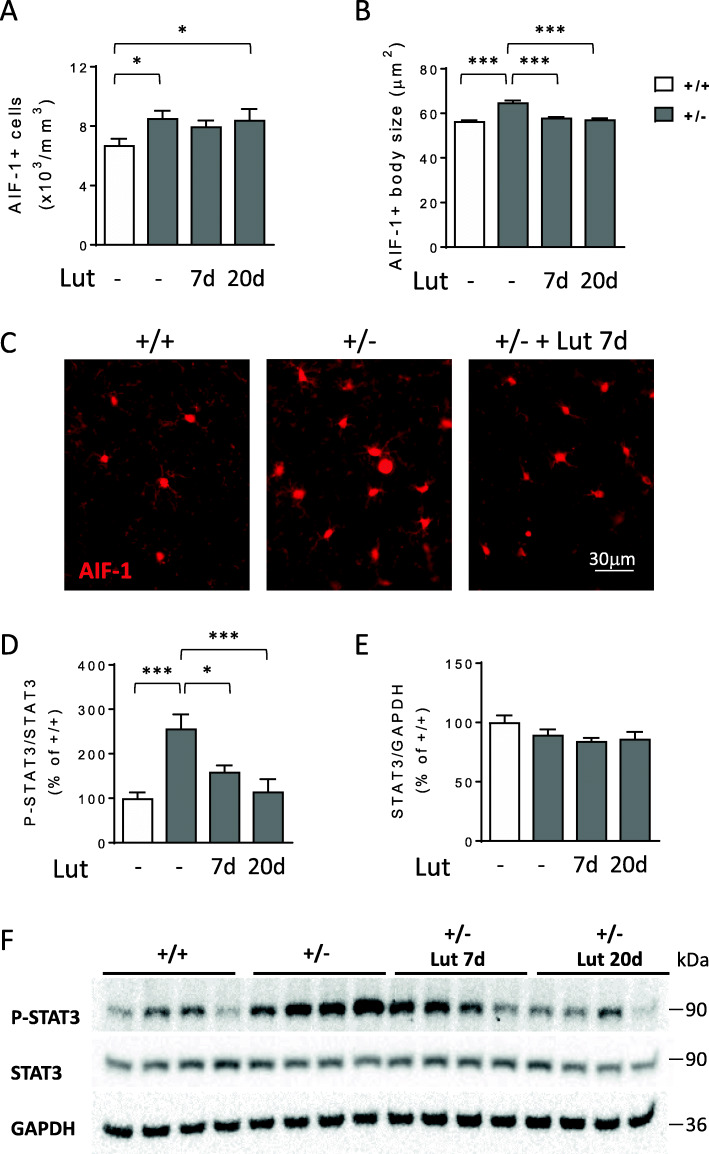
Effects of luteolin treatment on microglia activation in *Cdkl5* +/- mice. **A** Quantification of AIF-1-positive cells in somatosensory cortex from 3-month-old *Cdkl5* +/+ (n=5) and *Cdkl5* +/- (n=6) mice, and *Cdkl5* +/- mice daily treated with luteolin intraperitoneal injections (i.p. 10 mg/Kg) for 7 (Lut 7d, n=4) or 20 days (Lut 20d, n=4). **B** Mean AIF-1-cell body size of microglial cells in *Cdkl5* female mice as in **A**. **C** Representative fluorescence images of cortical sections processed for AIF-1 immunohistochemistry of a *Cdkl5* +/+ and a *Cdkl5* +/- mouse, and a 7-day luteolin-treated *Cdkl5* +/- mouse (+ Lut 7d). Values in **A** and **B** are presented as mean ± SEM. *p<0.05; ***p<0.001 (Fisher’s LSD test after one-way ANOVA). **D**, **E** Western blot analysis of P-STAT3 (Tyr 705), STAT3, and GAPDH levels in somatosensory cortex homogenates from untreated *Cdkl5* mice (+/+ n=4, +/- n=4) and luteolin-treated *Cdkl5* +/- mice as in **A**. Histograms show P-STAT3 (Tyr 705) protein levels normalized to corresponding total STAT3 protein levels in **D**, and STAT3 levels normalized to GAPDH in **E**. Data are expressed as percentages of *Cdkl5* +/+ mice. Values are presented as means ± SEM; *p<0.05; ***p< 0.001 (Fisher’s LSD test after one-way ANOVA). **F** Example of immunoblots from 4 animals of each experimental group

Luteolin, a naturally occurring polyphenolic flavonoid, is a potent microglia inhibitor that possesses antioxidant, anti-inflammatory, and neuroprotective effects both in vitro and in vivo [42, 43]. Since the majority of CDD patients are heterozygous females [44], we tested the efficacy of an in vivo treatment with luteolin on microglial overactivation in the heterozygous female mouse model of CDD (+/-). Three-month-old *Cdkl5* KO female mice (+/-) were injected daily with luteolin (i.p. 10 mg/Kg) for 7 or 20 days. While both short- and long-term treatments with luteolin did not affect microglia cell number in the cortex of *Cdkl5* +/- mice (Fig. 4A, C), a 7-day treatment was sufficient to recover microglia body size at the wild-type level in both cortex (Fig. 4B, C) and hippocampus (Fig. S3).

In brain homogenates of *Cdkl5* +/- mice, we found significantly higher levels of phosphorylated STAT3, a key promoter of the microglial cell proinflammatory phenotype [45, 46], in comparison with their wild-type counterparts (Fig. 4D–F). A 7-day treatment with luteolin restored phospho-STAT3 levels to those of wild-type mice (Fig. 4D, F). No differences in total STAT3 levels were observed in treated or untreated *Cdkl5* +/- mice in comparison with their wild-type counterparts (Fig. 4E, F).

**Fig. 5:**
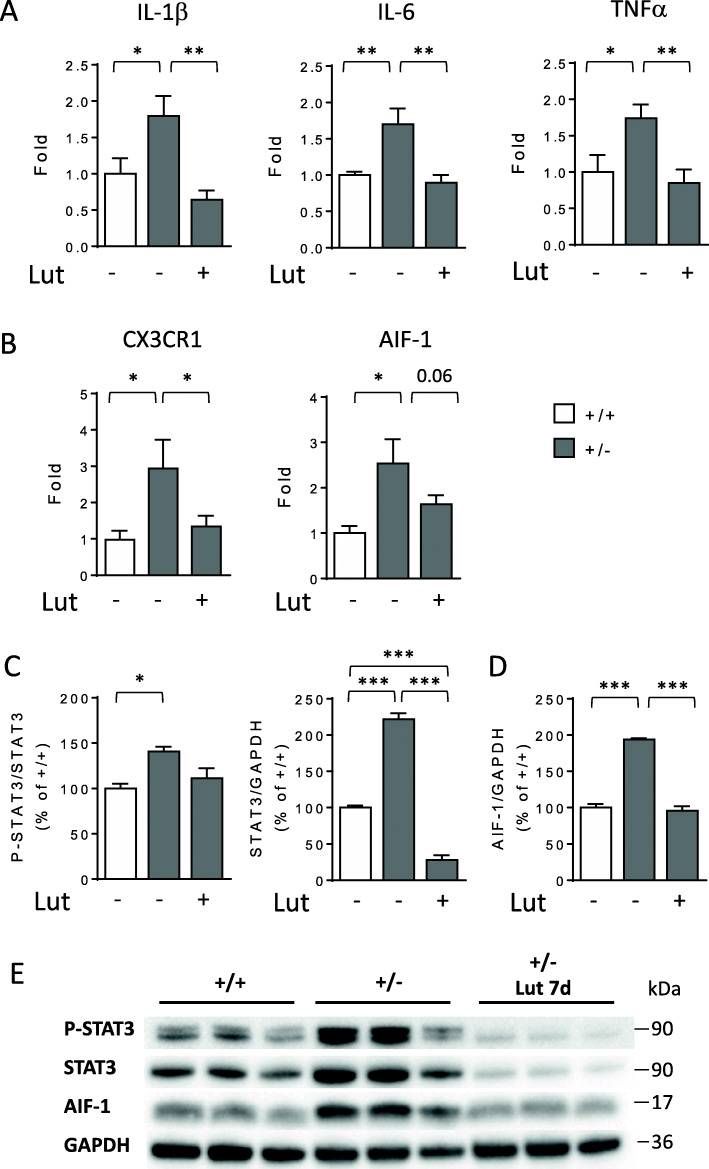
Effects of luteolin treatment on neuroinflammatory gene expression in microglial cells of *Cdkl5* KO mice. **A** Real-time qPCR analysis of interleukin 1 beta (IL-1β), interleukin 6 (IL-6), and tumor necrosis factor alpha (TNFα) gene expression in microglial cells isolated from the brain of 3-month-old *Cdkl5* +/+ (n=6) and *Cdkl5* +/- (n=5) mice and 7-day luteolin-treated *Cdkl5* +/- (n=6, Lut) mice. **B** Expression of CX3C chemokine receptor 1 (CX3CR1) and allograft inflammatory factor 1 (AIF-1) in microglial cells isolated from the brain of mice as in **A**. Data are given as fold change in comparison with microglial cells from *Cdkl5* +/+ mice. * p<0.05; ** p<0.01; (Fisher’s LSD test after one-way ANOVA). **C**, **D** Western blot analysis of P-STAT3 (Tyr 705), STAT3, AIF-1, and GAPDH levels in microglial cells isolated from the brains of untreated *Cdkl5* mice (+/+ n=4, +/- n=4) and 7-day luteolin-treated *Cdkl5* +/- mice (n=6). Histograms show P-STAT3 protein levels normalized to corresponding total STAT3 protein levels (**C**, left panel), STAT3 levels normalized to GAPDH (**C**, right panel), and AIF-1 levels normalized to GAPDH (**D**). **E** Example of immunoblots from the same experimental group as in **C**. The results in **C** and **D** are expressed as percentages of protein levels in *Cdkl5* +/+ microglial cells. Values are represented as means ± SEM of three technical replicates from the same sample; each sample has been obtained by mixing microglial cells purified from the brains of untreated *Cdkl5* +/+ and *Cdkl5* +/- mice (n=4) and 7-day luteolin-treated *Cdkl5* +/- mice (n=6). ***p < 0.001 (Fisher’s LSD test after one-way ANOVA)

To confirm microglia overactivation in the *Cdkl5* KO brain, we measured the expression of molecules involved in the microglial neuroinflammatory response in purified microglial cells, along with the levels of phospho-STAT3 and AIF-1. We found increased expression of IL-1β and IL-6 cytokines, TNFα, and microglial markers (CX3CR1 and AIF-1) in microglia cells from *Cdkl5* +/- mice in comparison with wild-types (Fig. 5A, B); the increased expression was recovered by treatment with luteolin (Fig. 5A, B). Similarly to what we observed in cortical extracts (Fig. 4D–F), we found an increase in P-STAT3 levels in microglial cells of *Cdkl5* +/- mice, but higher levels of total STAT3 in comparison with wild-type microglia (Fig. 5C, E). A 7-day treatment with luteolin drastically reduced total STAT3 levels in microglia cells (Fig. 5C, E), and, accordingly, the amount of STAT3 in the active form (Fig. 5C, E). Similarly, AIF-1 levels were higher in microglia cells from *Cdkl5* +/- mice and returned to the control value after treatment with luteolin (Fig. 5D, E).

### Treatment with luteolin restores survival and maturation of newborn cells in the dentate gyrus of *Cdkl5* +/- mice

**Fig. 6:**
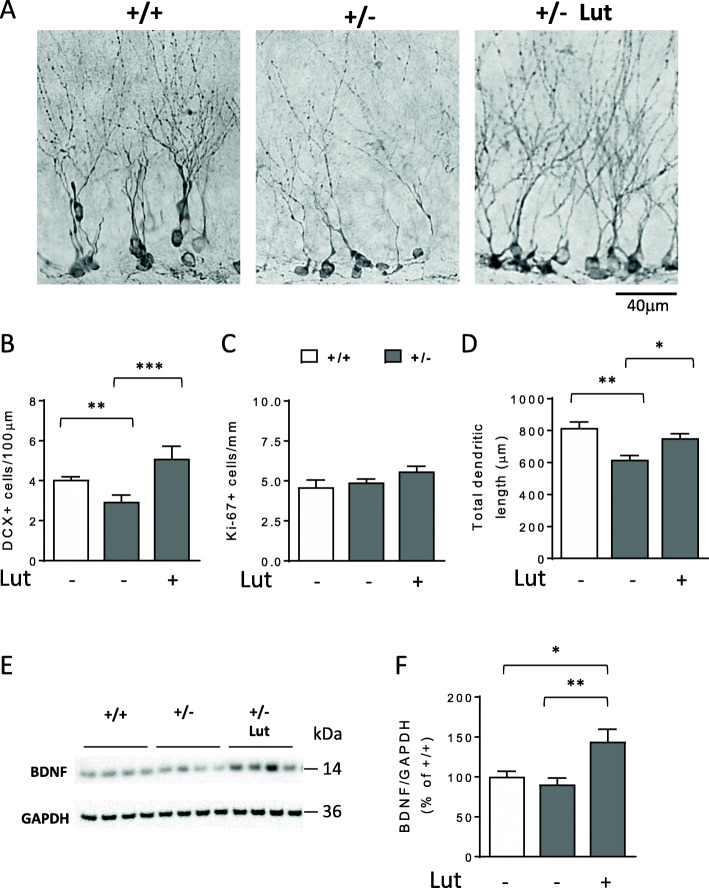
Effect of luteolin treatment on survival and maturation of postmitotic granule neurons in *Cdkl5* KO mice. **A** Examples of sections processed for DCX immunostaining from the dentate gyrus (DG) of an untreated wild-type (+/+), a heterozygous *Cdkl5* KO female (+/-), and a 7-day luteolin-treated heterozygous *Cdkl5* KO female (10 mg/Kg; +/- Lut 7d) mouse. **B** Number of DCX-positive cells in the DG of untreated *Cdkl5* +/+ (n=6) and *Cdkl5* +/- (n=6) mice, and 7-day luteolin-treated Cdkl5 +/- mice (Lut +, n=4). Data are expressed as number of DCX positive cells per 100 μm of granule cell layer. **C** Number of Ki-67-positive cells in the DG of mice as in **B**. **D** Mean total dendritic length of CA1 pyramidal neurons of mice as in **B**. Values in **B**, **C**, and **D** are represented as means ± SEM. *p< 0.05, **p< 0.01 (Fisher’s LSD test after one-way ANOVA). **E**, **F** Western blot analysis of BDNF and GAPDH levels in somatosensory cortex homogenates from untreated *Cdkl5* mice (+/+ n=4, +/- n=4) and 7-day luteolin-treated *Cdkl5* +/- mice (+/- Lut7d, n=4). Examples of immunoblot in **E**. Histogram in **F** shows mature BDNF protein levels normalized to GAPDH. Data are expressed as a percentage of untreated *Cdkl5* +/+ mice. Values are represented as means ± SEM of two technical replicates; *p<0.05;**p < 0.01 (Fisher’s LSD test after one-way ANOVA)

Loss of Cdkl5 impairs survival and maturation of newborn hippocampal neurons [11, 16]. In order to evaluate the efficacy of in vivo treatment with luteolin on the survival rate of new neurons, we assessed the number of doublecortin (DCX)-positive cells in the dentate gyrus (DG) of untreated *Cdkl5* +/- and *Cdkl5* +/+ mice and *Cdkl5* +/- mice treated with luteolin for 7 days. We found that treatment with luteolin restored the number of DCX-positive granule neurons in *Cdkl5* +/- mice (Fig. 6A, B). To determine whether increased proliferation rate underlies the positive effect of luteolin on newborn neuronal number, we counted proliferating cells immunostained for Ki-67, an endogenous marker of actively proliferating cells. We found that luteolin-treated *Cdkl5* +/− mice had the same number of Ki-67 labeled cells as untreated *Cdkl5* +/− and *Cdkl5* +/+ mice (Fig. 6C). This evidence indicates that the higher number of DCX-positive cells in treated *Cdkl5* +/− mice is not due to an increase in proliferation rate.

Importantly, luteolin treatment improved impaired dendritic development in *Cdkl5* +/- mice. Quantification of the dendritic tree of DCX positive cells showed that *Cdkl5* +/− mice had a shorter dendritic length than wild-type mice (Fig. 6A, D); a 7-day treatment with luteolin improved this defect (Fig. 6A, D).

It was reported that luteolin treatment increases brain levels of the brain-derived neurotrophic factor (BDNF) [33], which is necessary for neuronal survival and maturation [47]. We examined mature BDNF levels in cortical homogenates of *Cdkl5* +/- and *Cdkl5* +/+ mice and in *Cdkl5* +/- mice following administration of luteolin for 7 days. While levels of BDNF were similar in *Cdkl5* +/- and *Cdkl5* +/+ mice treated with the vehicle, treatment with luteolin increased levels of BDNF by about 50% (Fig. 6E, F).

### Inhibition of microglia overactivation restores survival of CA1 hippocampal neurons in *Cdkl5* +/- mice

**Fig. 7:**
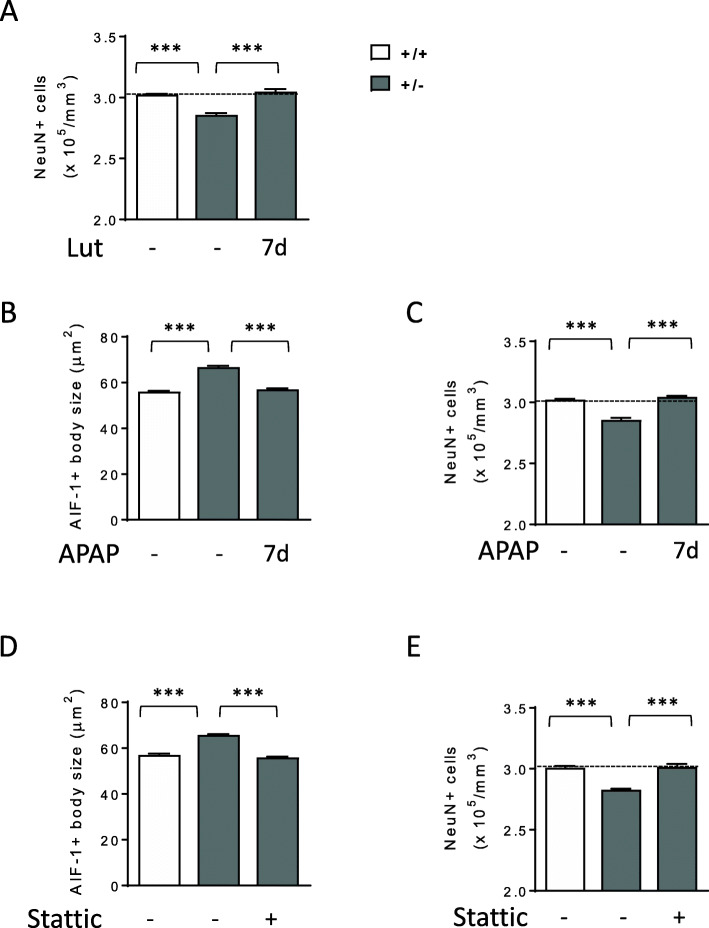
Effect of inhibition of microglia activation on hippocampal neuron survival in *Cdkl5* +/- mice. **A** Quantification of NeuN positive cells in CA1 layer of hippocampal sections from untreated *Cdkl5* mice (+/+ n=3, +/- n=3) and 7-day luteolin-treated *Cdkl5* +/- mice (+/- Lut7d, n=4). **B** Mean AIF-1-cell body size of microglial cells in the hippocampus of vehicle-treated 3-month-old *Cdkl5* +/+ (n=5) and *Cdkl5* +/- (n=6) mice and from *Cdkl5* +/- mice injected with acetaminophen (APAP; i.p. 100 mg/Kg) every day for 7 days (n=4). **C** Quantification of NeuN positive cells in CA1 layer of hippocampal sections from mice treated as in **B**. **D** Mean AIF-1-cell body size of microglial cells in the hippocampus of vehicle-treated 3-month-old *Cdkl5* +/+ (n=4) and *Cdkl5* +/- (n=4) mice and from *Cdkl5* +/- mice injected with Stattic (i.p. 20 mg/Kg), every other day for 7 days (n=4). **E** Quantification of NeuN positive cells in CA1 layer of hippocampal sections from mice treated as in **D**. The results in **A**–**E** are represented as means ± SEM. ***p<0.001 (Fisher’s LSD test after one-way ANOVA)

To further assess the efficacy of luteolin treatment on neuron survival, we evaluated the density of NeuN-positive cells in the CA1 layer of the hippocampus of *Cdkl5* +/- mice. Similarly to male *Cdkl5* -/Y mice [21], female *Cdkl5* +/- mice showed a low number of NeuN-positive pyramidal neurons in the CA1 layer compared with wild-type mice (Fig. 7A). Luteolin treatment restored neuronal number in *Cdkl5* +/- mice (Fig. 7A).

To confirm the beneficial effect of microglia inhibition on neuron viability, we treated *Cdkl5* +/- mice with acetaminophen (APAP), a main Cox2 inhibitor that reduces PGE2 production and suppresses microglial activation and proinflammatory cytokines [48, 49]. A 7-day treatment with APAP (100 mg/Kg) restored microglia body size and neuron survival in the hippocampus of *Cdkl5* +/- mice (Fig. 7B, C) to the levels of those found in wild-type mice.

Furthermore, we assessed the effect of treatment with Stattic, a selective inhibitor of STAT3 activation [34, 50, 51]. A 7-day in vivo inhibition of STAT3 with Stattic (20 mg/Kg) recovered microglia body size in the hippocampus of *Cdkl5* +/- mice (Fig. 7D). Accordingly, with the inhibition of microglia overactivation, we found a restoration of neuron survival in Stattic-treated *Cdkl5* +/- mice (Fig. 7E).

Overall, these results suggest the detrimental role of microglia overactivation in *Cdkl5* KO neuronal survival.

### Microglia activation with age in *Cdkl5* +/- mice

**Fig. 8:**
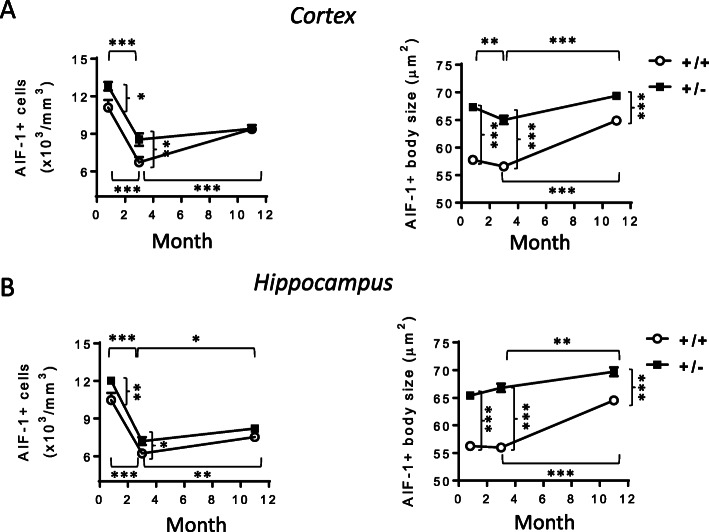
Assessment of microglia activation in different life stages of *Cdkl5* +/- mice. **A**, **B** Quantification of the number of AIF-1-positive cells (on the left) and mean AIF-1-cell body size (on the right) of microglial cells in somatosensory cortex (**A**) and hippocampal sections (**B**) from young (20-day-old; +/+ n=5; +/- n=6), adult (3-month-old; +/+ n=5; +/- n=6), and middle-aged (11-month-old; +/+ n=4; +/- n=4) *Cdkl5* mice. The results in **A** and **B** are presented as means ± SEM. * p<0.05; ** p<0.01; ***p<0.001 (Fisher’s LSD test after two-way ANOVA)

To assess whether microglia overactivation is already present at an early stage of life and to monitor its evolution with age, we analyzed microglial cell status in the brains of *Cdkl5* KO mice at different developmental stages (young 20-day-old, adult 3-month-old, and middle-aged 11-month-old mice). We found that an increase in microglial cell number and soma size was already present in the cortex and hippocampus of young *Cdkl5* +/- mice compared to their wild-type counterparts of the same age (Fig. 8A, B). A significant decrease in the density of microglial cells with age was present in both wild-type and *Cdkl5* +/- mice compared to their 20-day-old counterparts (Fig. 8A, B). Surprisingly, while the difference in the number of microglial cells was maintained between *Cdkl5* +/- and wild-type mice at 3 months of age, in middle-aged mice, there was no longer a difference (Fig. 8A, B). In contrast, increased microglial body size in *Cdkl5* +/- mice was present in all three age groups compared to their wild-type counterparts of the same age (Fig. 8A, B). Interestingly, an age-dependent worsening of microglial activation, and, therefore, microglial body size, was observed in both middle-aged *Cdkl5* +/- and *Cdkl5* +/+ mice (Fig. 8A, B).

### Luteolin treatment restores neuron survival in middle-aged *Cdkl5* +/- mice

Microglial cell changes compatible with their activation have been documented in aging [52, 53], and it has been suggested that they contribute to the brain decline in pathological conditions [54, 55]. Recent evidence showed an age-dependent decreased hippocampal neuron survival in middle-aged *Cdkl5* KO mice, paralleled by an increased cognitive decline [21].

**Fig. 9:**
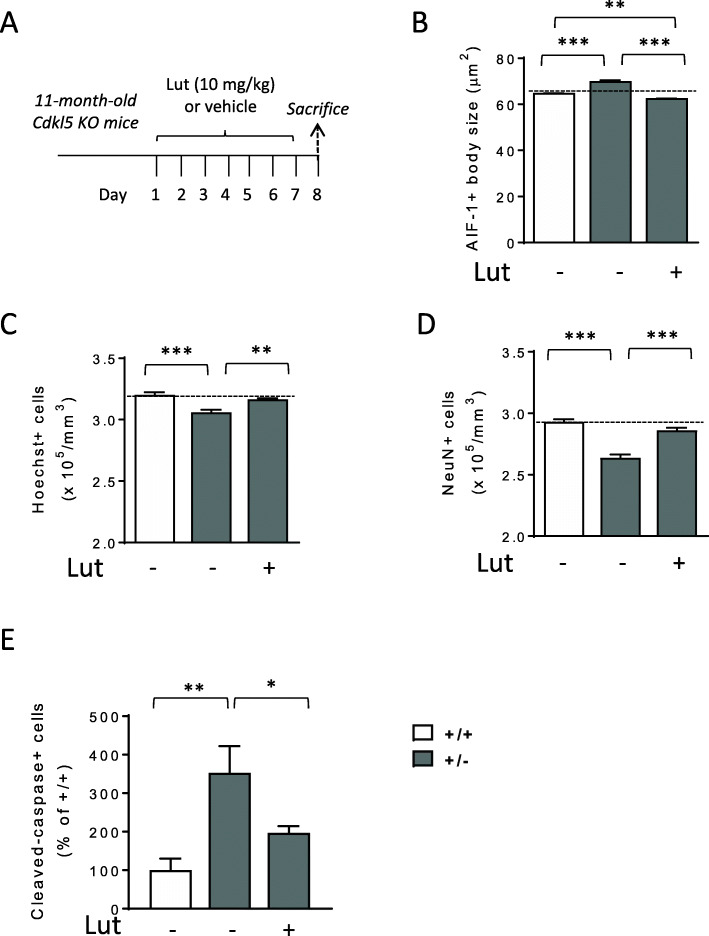
Effect of luteolin treatment in middle-aged *Cdkl5* +/- mice. **A** Schedule of treatment. Middle-aged (11-month-old) *Cdkl5* mice were treated with vehicle or luteolin for 7 days. Mice were sacrificed 1 day after the end of treatment. **B** Mean AIF-1-cell body size of microglial cells in the hippocampus of vehicle-treated (+/+ n=4; +/- n=4) and luteolin-treated (+/- n=4, Lut) middle-aged *Cdkl5* mice. **C**, **D** Quantification of Hoechst-positive cells (**C**) and NeuN positive cells (**D**) in CA1 layer of hippocampal sections from mice treated as in **C**. **E** Number of cleaved caspase-3 positive cells in the hippocampus of middle-aged (11-month-old) *Cdkl5* mice treated as in **A**. Data are given as a percentage of NMDA-treated *Cdkl5* +/+ mice. The results in **B**, **C**, **D**, and **E** are presented as means ± SEM. * *p*<0.05; ** *p*<0.01; ***p<0.001 (Fisher’s LSD test after one-way ANOVA)

To explore the possibility that microglia overactivation in aged *Cdkl5* KO mice could underlie the higher neuronal loss, we assessed the efficacy of treatment with luteolin in counteracting neuronal loss in 11-month-old *Cdkl5* +/- mice (Fig. 9A). Treatment with luteolin reduced microglia body size in the hippocampus of middle-aged *Cdkl5* +/- mice at even lower levels than those of wild-type mice of the same age (Fig. 9B). Importantly, the reduced number of Hoechst-positive nuclei (Fig. 9C) and NeuN-positive cells (Fig. 9D), and the increased number of apoptotic cleaved caspase-3 positive cells (Fig. 9E) in middle-aged *Cdkl5* +/- mice were strongly improved by treatment with luteolin (Fig. 9C–E).

### Treatment with luteolin prevents NMDA-induced seizure persistence and excitotoxicity in the hippocampus of *Cdkl5* +/- mice

**Fig. 10:**
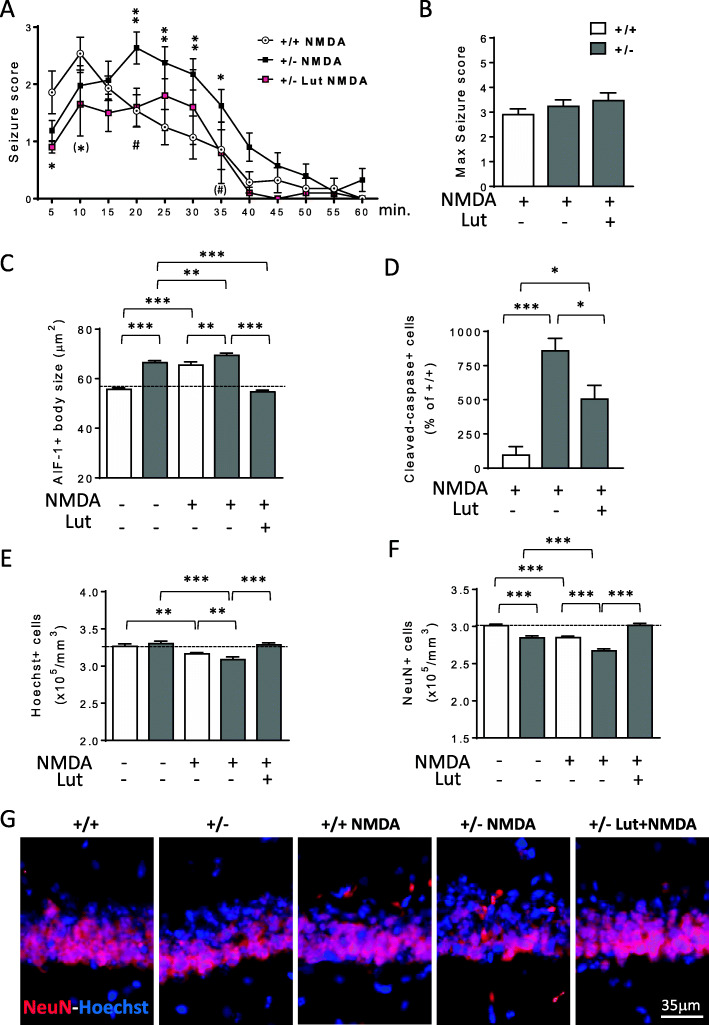
Effect of luteolin treatment on NMDA-induced excitotoxicity in the hippocampus of *Cdkl5* +/- mice. ***A*** Graph represents seizure score of 3-month-old *Cdkl5* +/+ (n=14) and *Cdkl5* +/- (n=21) mice treated with a single intraperitoneal injection of NMDA (60 mg/kg) and of *Cdkl5* +/- (n=10) mice treated with NMDA after 7 days of luteolin treatment. The results are presented as means ± SEM. (*) p=0.056; * p<0.05; ** p<0.01 as compared to the NMDA-treated *Cdkl5* +/+ condition; (#) p=0.055; #p < 0.05; as compared to the NMDA-treated *Cdkl5* +/- condition (Fisher’s LSD test after two-way ANOVA). **B** Histogram shows the mean of the maximum seizure score in mice treated as in **A**. **C** Mean AIF-1-cell body size of microglial cells in the hippocampus of *Cdkl5* +/+ (n=5) and *Cdkl5* +/- (n=6) mice treated with vehicle only, in *Cdkl5* +/+ (n=3) and *Cdkl5* +/- (n=3) mice treated with vehicle and NMDA and in *Cdkl5* +/- (n=3) mice pre-treated for 7 days with luteolin before NMDA injection. Mice were sacrificed 8 days after NMDA treatment. **D** Number of cleaved caspase-3 positive cells in the hippocampus of *Cdkl5* +/+ (n=3) and *Cdkl5* +/- (n=4) mice treated with vehicle and NMDA, and in *Cdkl5* +/- (n=6) mice pre-treated for 7 days with luteolin before NMDA injection. Mice were sacrificed 24 h after NMDA treatment. Data are given as a percentage of NMDA-treated *Cdkl5* +/+ mice. **E**, **F** Quantification of Hoechst-positive cells (**E**), and NeuN positive cells (**F**) in CA1 layer of hippocampal sections from mice treated as in **B**. The results in **B**, **E**, and **F** are presented as means ± SEM. * p<0.05; ** p<0.01; ***p<0.001 (Fisher’s LSD test after one-way ANOVA). **G** Representative fluorescent images of hippocampal sections of mice treated as in **B** immunostained for NeuN and counterstained with Hoechst

Increasing evidence supports a link between inflammation and epilepsy [56]. To investigate whether microglia overactivation predisposes induced seizure-like events and has a causative role in the increased neuronal susceptibility to excitotoxic stress in *Cdkl5* KO mice [19, 20], we pre-treated *Cdkl5* +/- mice for 7 days with luteolin before NMDA (60mg/kg) administration (Fig. S4A). Seizure grades were scored during a 60-min observation period after NMDA injection, and mice were sacrificed 1 day or 8 days afterwards (Fig. S4A). We found that *Cdkl5* +/- mice showed a different trend of seizure persistence (Fig. 10A), but no difference in seizure severity (Fig. 10B), compared to the NMDA-treated wild-type counterparts. We found that, while in wild-type mice the highest scores occur in the first 5–15 min, *Cdkl5* +/- mice showed a persistence in the higher seizure scores up to 30–35 min after NMDA administration. In addition, seizure freedom is achieved more slowly in *Cdkl5* +/- mice in comparison with wild-type mice. Importantly, pre-treatment with luteolin shortens seizure persistence in NMDA-treated *Cdkl5* +/- mice (Fig. 10A), suggesting that microglia activation is involved in the susceptibility to prolonged seizures that characterizes *Cdkl5* KO mice.

As expected, microglia activation increased in the hippocampus of both *Cdkl5* +/- and *Cdkl5* +/+ mice after NMDA treatment (Fig. 10C). Nevertheless, after NMDA stimulation, the somal volume of microglial cells in *Cdkl5* +/- mice was higher than that of NMDA-treated wild-type mice (Fig. 10C). Importantly, luteolin treatment was able to counteract both basal and NMDA-induced microglial activation in *Cdkl5* KO mice, bringing microglial soma size back to that of the untreated wild-type mouse condition (Fig. 10C).

Neuronal death was assessed 1 day after NMDA administration using immunohistochemistry for cleaved caspase-3 and 8 days after using Hoechst staining and immunohistochemistry for NeuN. In the CA1 layer of the hippocampus, NMDA-treated *Cdkl5* +/- mice showed a higher number of cleaved caspase-3 positive cells (Fig. 10D and Fig. S4B) and a lower number of Hoechst-positive nuclei (Fig. 10E) and NeuN-positive cells (Fig. 10F,G) in comparison with NMDA-treated wild-type mice, indicating increased cell death in *Cdkl5* +/- mice after the excitotoxic stimulus. Importantly, pre-treatment with luteolin reduced cell death at 24 h after NMDA treatment in *Cdkl5* +/- mice (Fig. 10D and Fig. S4B), thus preventing neuronal loss in the hippocampal CA1 region (Fig. 10E–G).

As both pyramidal neurons and GAD67-positive interneurons are NeuN-positive [57], to exclude an effect of the treatments on the number of interneurons, we evaluated the number of GAD67-positive cells in the hippocampus of treated *Cdkl5* +/- mice (Fig. S4C). We found no difference in the number of inhibitory interneurons among NMDA-treated *Cdkl5* +/+, NMDA-treated *Cdkl5* +/-, and luteolin/NMDA-treated *Cdkl5* +/- mice (Fig. S4C), indicating that NMDA-induced excitotoxicity does not affect inhibitory neuron viability and that pre-treatment with luteolin does not promote their survival. Similarly, we found no differences in the number of GFAP-positive astrocytes (Fig. S4D).

## Discussion

The lack of effective therapies for CDD stresses the urgency with which pathogenic mechanisms underlying the disorder need to be identified. Our results highlight, for the first time, the presence of a generalized status of microglia overactivation in the brain of a mouse model of CDD. We found alterations in microglial cell morphology and number, increased levels of AIF-1 and proinflammatory cytokines, and increased STAT3 signaling in the brain of *Cdkl5* KO mice. Remarkably, treatment with luteolin (a natural anti-inflammatory flavonoid) is able to recover impaired neuronal survival and maturation in *Cdkl5* KO mice, suggesting that a hyperactive state of microglia plays a causative role in the CDD phenotype.

Recently, a cytokine dysregulation, proportional to clinical severity and redox imbalance, was found in children affected by CDD [31, 32]. Results included increased tumor necrosis factor (TNF)-α, interleukin (IL)-1β, and interleukin (IL)-6 in the peripheral blood of children with CDD. These inflammatory cytokines could signal inflammatory changes in the brain that could, in turn, greatly impact neurodevelopment and neural function in CDD. Our finding of an overactivation of microglial cells with increased TNF-α, IL-1β, and IL-6 levels in the brain of *Cdkl5* KO mice is in line with the results observed in CDD patients and suggests the involvement of neuroinflammatory processes in the pathophysiology of CDD.

Microglia activation, associated with an increase in cell body size, as well as cytokine alterations in the peripheral blood, has been recently described in neurodevelopmental disorders such as autism spectrum disorders (ASDs), Down syndrome, and Rett syndrome [28–30, 58, 59]. Similarly to Rett syndrome [60], the mechanism by which absence of *Cdkl5* induces microglia overactivation appeared to be non-cell autonomous. By using *Emx1* KO mice, a conditional *Cdkl5* KO mouse model which does not carry *Cdkl5* deletion in microglial cells [6, 37], we found that microglia overactivation is independent of microglia-specific loss of Cdkl5 expression. This finding suggests that microglial activation in the *Cdkl5* KO brain may be attributable to neuronal loss of Cdkl5. Accumulating evidence indicates the presence of bidirectional microglia-neuron communication in the healthy and diseased brain [61]. In the healthy brain, microglia exhibit an actively repressed “surveying” phenotype that is dependent on a dynamic crosstalk between microglia and neurons [62]. It has been proposed that the removal of this neuronal-derived inhibitory control represents a type of danger signal for microglia, indicating that neuronal function is impaired and leads to alterations in microglia morphology and function. The chronic activation of microglia may, in turn, cause reduced neuronal maturation and survival through the release of potentially cytotoxic molecules such as proinflammatory cytokines [23, 24]. Our finding that inhibition of microglia overactivation by luteolin restores survival and maturation of newborn neurons in the hippocampal dentate gyrus of *Cdkl5* KO mice is in line with this hypothesis. Though luteolin treatment had no effect on the number of microglial cells, it restored microglia body size and shape, and, importantly, proinflammatory cytokines and P-STAT3 levels. The evidence that in microglia cells not only STAT3 phosphorylation levels but also total STAT3 levels are higher in *Cdkl5* +/- mice may depend on the increased STAT3 gene expression in response to proinflammatory cytokines [63]. On the other hand, luteolin-dependent accelerated ubiquitin-dependent degradation of the Tyr705-phosphorylated STAT3 protein [64, 65] can underlie the drastically reduced STAT3 levels in luteolin-treated *Cdkl5* +/- mice. However, at present, we have no indication as to why the effect on STAT3 degradation is evident in microglial cells and not in brain extracts of luteolin-treated *Cdkl5* +/- mice. We can hypothesize a different regulatory effect on STAT3 stability at the neuronal level.

Differently from a recent study that demonstrated the effect of luteolin treatment in increasing cell proliferation in a mouse model of Down syndrome [33], we did not observe an increased number of Ki-67-positive cells in the hippocampal dentate gyrus of *Cdkl5* KO mice. This discrepancy can be explained by a selective effect of luteolin on the Ts65Dn mouse, as the authors themselves did not observe a luteolin-dependent proliferation increase in wild-type mice. The pro-survival effect of microglial inhibition was confirmed by the restoration of the age-dependent decreased hippocampal neuron survival in middle-aged *Cdkl5* KO mice. This is in agreement with recent studies that suggest that luteolin plays a role in counteracting age-induced microglia activation in aged mice [66, 67].

The neuroprotective effect of luteolin might also be associated with its effect on increasing BDNF levels. Recent findings reported that luteolin increases the expression of BDNF in the cerebral cortex and hippocampus of mice [33, 68]. Similarly to these findings, we found increased BDNF levels in the brain of luteolin-treated *Cdkl5* KO mice. Since BDNF plays an important role in the survival and development of neurons [69], it is reasonable to hypothesize that, in *Cdkl5* KO mice, the luteolin-dependent activation of the BDNF pathways contributes to hippocampal neuronal survival and maturation. However, the evidence that other compounds (APAP and Stattic) are able to inhibit microglia overactivation by different mechanisms to those of luteolin [34, 49, 50, 70], positively affecting neuronal survival, further supports the detrimental role of microglia overactivation in *Cdkl5* +/- mice.

As previously reported in male *Cdkl5* KO mice [19, 20], we found here that hippocampal neurons of heterozygous female *Cdkl5* KO mice showed an increased vulnerability to excitotoxicity. Inflammatory processes, including activation of microglia and production of proinflammatory cytokines, such as TNF-α, IL-1β, and IL-6, are associated with excitotoxic stimuli [71, 72]. Similarly, we observed an increased microglial overactivation in response to NMDA treatment. Importantly, the microglia activation was higher in *Cdkl5* KO mice than in wild-type mice. Luteolin pre-treatment recovered the increased NMDA-induced cell death in hippocampal neurons of *Cdkl5* KO mice. Because the increased cell death of hippocampal neurons observed in response to NMDA treatment correlates with an increased microglial overactivation, the beneficial effect of luteolin in neuronal survival can be, at least partially, ascribed to the inhibition of microglia activation. Luteolin was observed to exert a similar neuroprotective activity against kainic acid-induced brain damage in mice [73].

Previously, we reported no difference between male *Cdkl5* -/Y and wild-type (+/Y) mice in seizure intensity following NMDA administration [20]. Here, we found that NMDA-treated heterozygous *Cdkl5* female mice showed increased seizure persistence, but no difference in seizure severity, compared to their wild-type counterparts, highlighting a difference between hemizygous male and heterozygous female *Cdkl5* KO mice in response to epileptic stimulus. This is in line with recent findings showing that only heterozygous female *Cdkl*5 KO mice are prone to developing spontaneous seizure activity [74, 75]. Interestingly, pre-treatment with luteolin partially restores NMDA-induced tonic-clonic seizure persistence. This effect may be ascribed to the increased release of proinflammatory cytokines by activated microglia cells which may lead to neuronal hyperexcitability [76–78]. It has been shown that epileptic seizures are induced in rats after intraventricular injection with an activated microglia-conditioned medium, suggesting that activation of microglia may also be an important process for the onset of epilepsy [79]. Accordingly, several studies have shown that pre-treatment with luteolin significantly reduces the frequency of pentylentetrazol-induced seizures in animal models of epilepsy [80–82].

## Conclusions

Microglia remain to be controversial cells within the CNS, with both beneficial and detrimental roles, especially in the context of disease pathology. Our finding that treatment with luteolin recovers neuronal survival and maturation in *Cdkl5* KO mice supports the theory that microglia exert a harmful action in the CDKL5-null brain. Recent evidence strongly supports the role of neuroinflammation in the pathophysiology of human drug-resistant epilepsies [83–85]. Since epileptic seizures are usually the first and most disabling symptoms of CDD patients, it may be hypothesized that luteolin has a beneficial effect on this feature. Therefore, the suppression of microglia-mediated inflammation should be considered as a possible therapeutic option for CDD.

## Supplementary Information

**Additional file 1:.** Supplementary materials.

**Additional file 2.**

## Data Availability

The datasets analyzed during the current study are available from the corresponding author on reasonable request.

## Abbreviations

AIF-1: Allograft inflammatory factor 1
ASD: Autism spectrum disorders
BDNF: Brain-derived neurotrophic factor
CDD: CDKL5 deficiency disorder
DCX: Doublecortin
DG: Dentate gyrus
GAD67: Glutamic acid decarboxylase 67
GFAP: Glial fibrillary acidic protein
IL-1β: Interleukin-1β
IL-6: Interleukin-6
NMDA: N-Methyl-D-aspartate
PSD-95: Postsynaptic density protein 95
STAT3: Signal transducer and activator of transcription 3
TNF-α: Tumor necrosis factor-α
