# Supplementary material for: Inhibition of microglia overactivation restores neuronal survival in a mouse model of CDKL5 deficiency disorder
Source: J Neuroinflammation. 2021 Jul 8;18:155. doi: 10.1186/s12974-021-02204-0 (PMC8265075; doi:10.1186/s12974-021-02204-0)
Supplement: Supplementary file 1 — Additional file 1:. Supplementary materials. [file 12974_2021_2204_MOESM1_ESM.pdf]

Gene		Primer sequence (*5'-3'*)
IL-1β	*Forward*	TGCCACCTTTTGACAGTGATG
	Reverse	TGATGTGCTGCTGCGAGATT
IL-6	*Forward*	CTCTGCAAGAGACTTCCATCCA
	Reverse	GACAGGTCTGTTGGGAGTGG
TNFα	*Forward*	TAGCCCACGTCGTAGCAAAC
	Reverse	GCAGCCTTGTCCCTTGAAGA
CX3CR1	*Forward*	TGCTTGCACATTGGGGAGACTGGA
	Reverse	AGGGAACGCTAAAGTCCTGGCTGA
AIF-1	*Forward*	GTCCTTGAAGCGAATGCTGG
	Reverse	CATTCTCAAGATGGCAGATC
mCdkl5	*Forward*	TGCAGACACAAGGAAACACATGA
	Reverse	TTTCCTGCTTGAGAGTGCGAA
CD11b	*Forward*	CCTTGTTCTCTTTGATGCAG
	Reverse	GTGATGACAACTAGGATCTT
NeuN	*Forward*	ACACACACACTCCATACTGAGG
	Reverse	GCTCTGGGCTCTCTGTTTGC

Supplementary Table 1. List of primers used for quantitative RT-PCR.

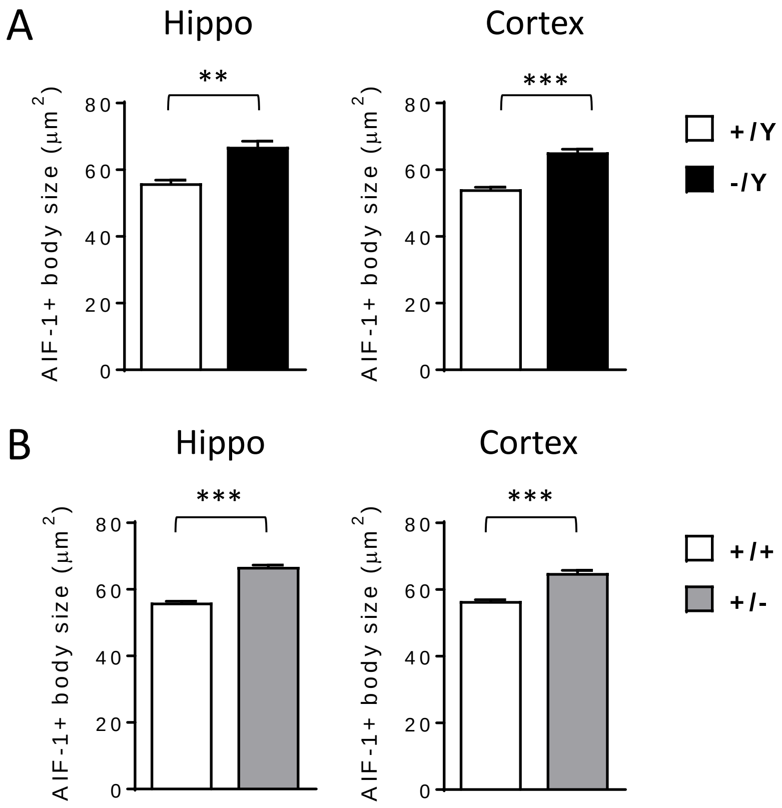

Supplementary Figure 1

Mean AIF-1-cell body size of microglial cells in hippocampal (Hippo) and somatosensory cortex (Cortex) sections from 3-month-old male (+/Y n=4, -/Y n=4; A), and female (+/+ n=5, +/- n=6; B) *Cdkl5* KO mice. The results in A and B are presented as means ± SEM. ** p<0.01; ***p<0.001 (two-tailed Student's t-test).

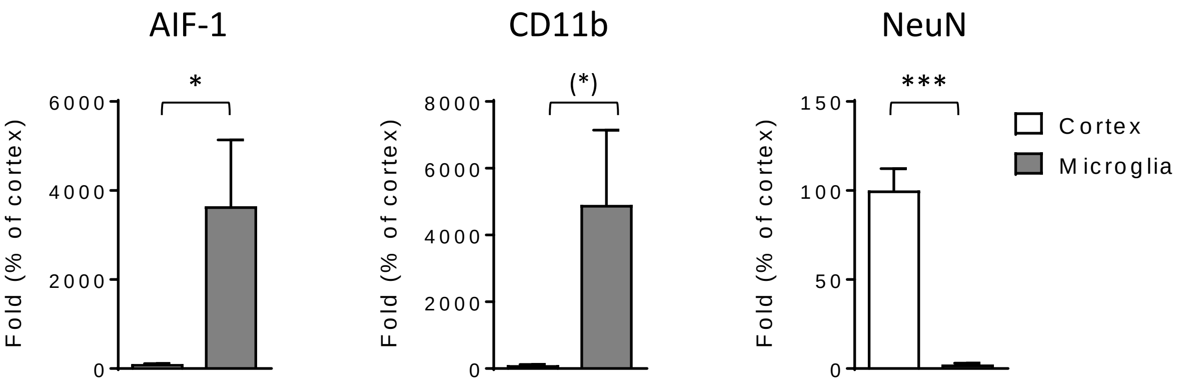

Supplementary Figure 2

Real-time qPCR analysis of Allograft inflammatory factor 1 (AIF-1), CD11b, and NeuN gene expression in the cortex of 3-month-old *Cdkl5* +/+ mice (n=6) and microglial cells purified from 3-month-old *Cdkl5* +/+ mice (n=6). Data are given as a percentage of *Cdkl5* +/+ mice cortical expression. The results are presented as means ± SEM. (*) $p=0.057$; * $p<0.05$; ***$p<0.001$ (two-tailed Student's t-test).

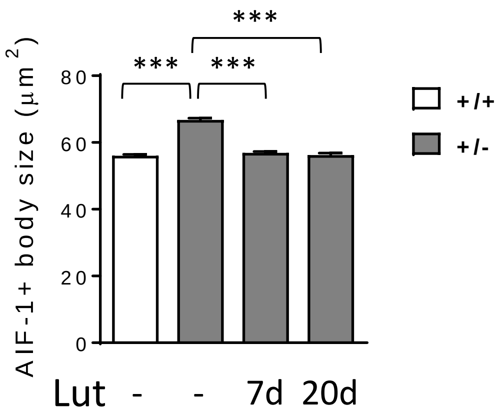

Supplementary Figure 3

Mean AIF-1-cell body size of microglial cells in the hippocampus of 3-month-old *Cdkl5* +/+ (n=5) and *Cdkl5* +/- (n=6) mice, and *Cdkl5* +/- mice daily treated with luteolin intraperitoneal injections (i.p. 10 mg/kg) for 7 (Lut 7d, n=4) or 20 days (Lut 20d, n=4). Values are represented as mean ± SEM. ***p<0.001 (Fisher's LSD test after one-way ANOVA).

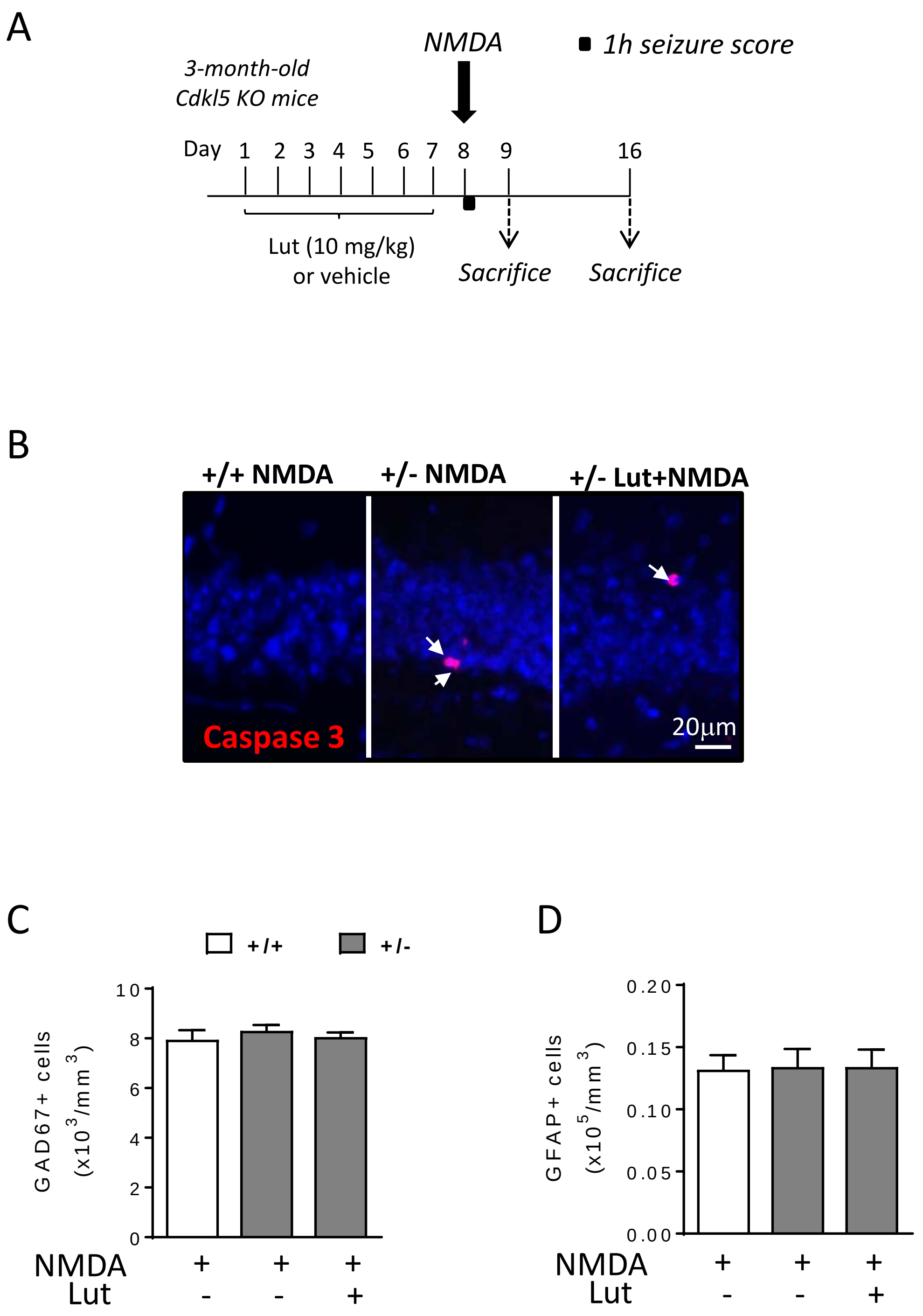

A
NMDA
1h seizure score
3-month-old
Cdkl5 KO mice
Day 1 2 3 4 5 6 7 8 9 16
Lut (10 mg/kg)
or vehicle
Sacrifice
Sacrifice
B
+/+ NMDA
+/- NMDA
+/- Lut+NMDA
Caspase 3
20µm
C
+/+
+/-
GAD67+ cells (x10^3/mm^3)
0 2 4 6 8 10
NMDA + + +
Lut - - +
D
GFAP+ cells (x10^5/mm^3)
0.00 0.05 0.10 0.15 0.20
NMDA + + +
Lut - - +

Supplementary Figure 4

A: Schematic view of *in vivo* treatments in 3-month-old *Cdkl5* KO female mice. Mice received a single intraperitoneal injection of NMDA (60 mg/kg) after 7 days of vehicle or luteolin treatment. Seizure grades were scored in a 60-minute observation period after NMDA injection (black square). Animals were sacrificed 1 day or 8 days after NMDA administration. **B**: Examples of cleaved caspase-3 positive cells (white arrows) in the hippocampus of a *Cdkl5* +/+ and a *Cdkl5* +/- mouse treated with vehicle and NMDA, and in a *Cdkl5* +/- mouse pre-treated for 7 days with luteolin before NMDA injection. Mice were sacrificed 24 h after NMDA treatment. **C,D**: Quantification of GAD67 (C) and GFAP (D) positive cells in CA1 layer of hippocampal sections from mice treated as in A and sacrificed 8 days after NMDA administration. Values in C,D are represented as means ± SEM. (Fisher's LSD test after one-way ANOVA).
