# Supplementary material for: Inhibition of microglia overactivation restores neuronal survival in a mouse model of CDKL5 deficiency disorder
Source: J Neuroinflammation. 2021 Jul 8;18:155. doi: 10.1186/s12974-021-02204-0 (PMC8265075; doi:10.1186/s12974-021-02204-0)
Supplement: Supplementary file 2 — Additional file 2. [file 12974_2021_2204_MOESM2_ESM.pdf]

*Supplementary information for reviewers*

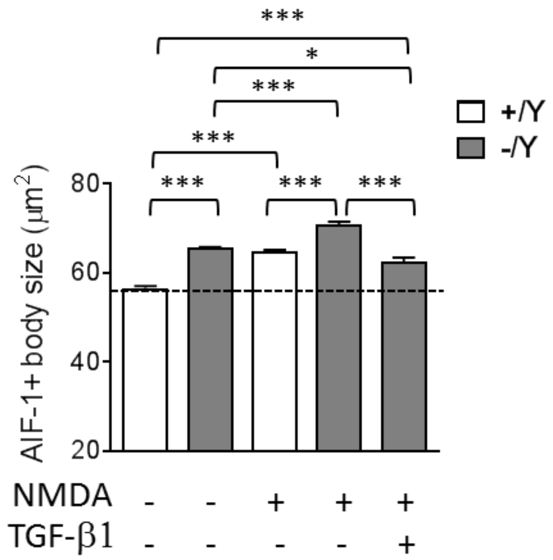


***Effect of TGF-β1 treatment on NMDA-induced microglia activation in the hippocampus of Cdkl5 KO mice.***

*10–12-week-old Cdkl5 +/Y and Cdkl5 −/Y mice were treated with vehicle only (n=2) or with an intraperitoneal injection of NMDA (60 mg/kg; n=4). To assess the effect of TGF-β1 treatment on NMDA-induced microglia activation, Cdkl5 −/Y (n=5) mice were intraventricularly injected with TGF-β1 (50 ng) 1 h after NMDA administration. Mice were sacrificed 24 h after NMDA treatment (Fuchs et al. 2019). The histogram shows the mean AIF-1-cell body size of microglial cells in the hippocampus of Cdkl5 mice treated as described above. The results are presented as means ± SEM. * $p<0.05$; ***$p<0.001$ (Fisher's LSD test after one-way ANOVA).*
